# Long-chain polyunsaturated fatty acid sources and evaluation of their nutritional and functional properties

**DOI:** 10.1002/fsn3.121

**Published:** 2014-06-29

**Authors:** Elahe Abedi, Mohammad Ali Sahari

**Affiliations:** Department of Food Science and Technology, Faculty of Agriculture, Tarbiat Modares UniversityTehran, Iran

**Keywords:** Docosahexaenoic acid, eicosapentaenoic acid, long-chain polyunsaturated fatty acids, nutritional and functional properties, sources, *α*-linolenic acid

## Abstract

Recent studies have clearly shown the importance of polyunsaturated fatty acids (as essential fatty acids) and their nutritional value for human health. In this review, various sources, nutritional properties, and metabolism routes of long-chain polyunsaturated fatty acids (LC-PUFA) are introduced. Since the conversion efficiency of linoleic acid (LA) to arachidonic acid (AA) and also α-linolenic acid (ALA) to docosahexaenoic acid (DHA) and eicosatetraenoic acid (EPA) is low in humans, looking for the numerous sources of AA, EPA and EPA fatty acids. The sources include aquatic (fish, crustaceans, and mollusks), animal sources (meat, egg, and milk), plant sources including 20 plants, most of which were weeds having a good amount of LC-PUFA, fruits, herbs, and seeds; cyanobacteria; and microorganisms (bacteria, fungi, microalgae, and diatoms).

## Introduction

**Figure 1:**
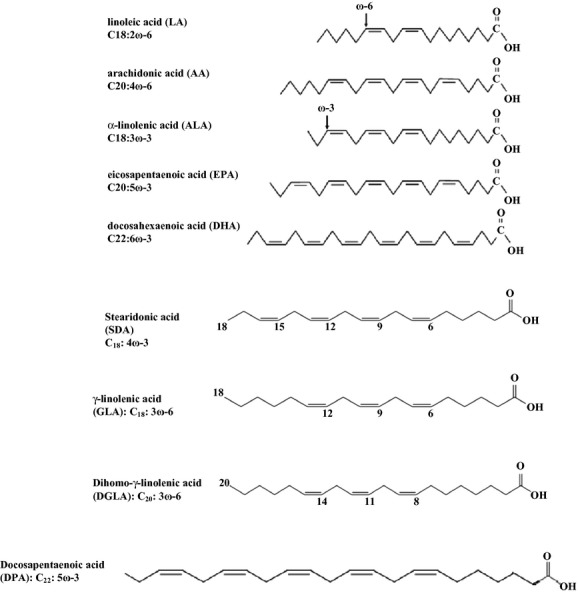
Long-chain polyunsaturated fatty acid structure.

Long-chain polyunsaturated fatty acids (LC-PUFAs) are fatty acids with 18–20 carbons or more, which can be categorized into two main families — *ω*6 (n-6) and *ω*3 (n-3) — depending on the position of the first double bond from the methyl end group of the fatty acid (Venegas-Calerón et al. 2010). Main n-3 LC-PUFA in food sources are *α*-linolenic acid (ALA) (18:3 Δ9, 12, 15), docosahexaenoic acid (DHA) (22:6 Δ4, 7, 10, 13, 16, 19), eicosapentaenoic acid (EPA) (20:5 Δ5, 8, 11, 14, 17), and docosapentaenoic acid (DPA) (22:5 Δ7, 10, 13, 16, 19), and n-6 LC-PUFA include linoleic acid (LA) (18:2 Δ9, 12) and arachidonic acid (AA) (20:4 Δ5, 8, 11, 14). Some intermediate products such as di-homo-*γ*-linolenic acid (DHGLA; 20:3 Δ8,11,14) and *γ*-linolenic acid (GLA; 18:3 Δ6,9,12) (for n-6 production), and stearidonic acid (SDA; 18:4 Δ6,9,12,15) and eicosatetraenoic acid (ETA; 20:4 Δ8,11,14,17) (for n-3 production) are created during LC-PUFA production. The molecular structure of the fatty acids mentioned above is shown in Figure 1.

As mammals cannot synthesize LA and ALA from the precursor oleic acid, the conversion efficiency of LA and ALA to AA, DHA, and EPA are low and hence direct uptake appears to be significantly more effective.

Although fish is the main source of LC-PUFAs, there are several limitations in using fish oil as a source of supply of these essential fatty acids. The limitations involve: (1) teratogen, carcinogen, and mutagen contaminants including dichloro diphenyl trichloro ethane (DDT) and dioxin-like polychlorinated biphenyls; (2) noncarcinogen contaminants such as methyl mercury, heavy metals (Pb, Cr, Hg, Cd, and As), and antibiotics (Sidhu 2003; Foran et al. 2005; Perveen et al. 2006; Li and Hu 2009); (3) fish oils possess undesirable odors, flavors, and tastes; (4) stability problems; (5) high cost and difficulty of purification due to the low value of DHA and EPA fatty acids recovered from fish; and (6) populations such as pregnant and lactating women and young children having higher risks for marine pollution (Foran et al. 2005; Park and Johnson 2006). These items combined with the decreasing fish stocks throughout the world have raised concerns about these resources. Moreover, since it is shown that LC-PUFAs are unhealthy, different attempts have been carried out to supply LC-3PUFA with alternative safe sources. In this review, the metabolism of LC-PUFAs in humans, eukaryotes, plants, algae, and protists and their nutritional function was evaluated. Other sources of LC-PUFA including animals, plants, and microorganisms were then investigated.

## Nutrition Functional of LC-PUFA

LC-PUFA is a precursor eicosanoid composed of prostaglandins and thromboxane. LC-PUFA plays an important role in immune system regulation, blood clots, neurotransmitters, cholesterol metabolism, and structure of membrane phospholipids in the brain and the retina.

Some nutritional functions of LC-PUFA are as follows:

1. The inhibition of synthesis of vasoaggressive low-density lipoprotein (LDL) (Steffens and Wirth 2005) and acceleration of LDL elimination (Steffens and Wirth 2005). However, they do not have any influence on the vasoprotective high-density lipoprotein (HDL) or even on the enhancement of HDL production (Steffens and Wirth 2005);
2. Reduction in platelet, prolongation of bleeding time, and reduction in blood pressure Steffens and Wirth 2005);
3. LC-PUFA *ω*3 also have beneficial effects on diseases other than those of the heart or blood vessels. The diseases include skin disease (Steffens and Wirth 2005), asthma (Lands 1986), arthritis (Kremer et al. 1988), nephritis (Thais and Stahl 1987), lupus erythematosus (Kelley et al. 1985), and multiple sclerosis (Bates et al. 1989);
4. A major component of most biological membrane phospholipids and important in membrane structure and function (Li and Hu 2009);
5. DHA is highly concentrated in the retina and the brain in humans and other mammals, and is essential for normal visual and brain function (Li and Hu 2009);
6. Contributes to membrane fluidity (membrane order), which can influence the function of membrane receptors such as rhodopsin (Li and Hu 2009);
7. Regulation of membrane-bound enzymes (Na/K-dependent ATPase) and plays a role in signal transduction via having effects on inositol phosphates, diacylglycerol (DAG), and protein kinase C (Li and Hu 2009);
8. DHA directly influences neurotransmitter biosynthesis, signal transduction, uptake of serotonin, binding of *β*-adrenergic and serotonergic receptors, and mono amine oxidase activity (Li and Hu 2009);
9. Regulation of eicosanoid production from AA, whereby EPA competes with AA to produce various eicosanoids such as three series of prostaglandins, prostacyclin, and thromboxane; and five series of leukotrienes (Li and Hu 2009);
10. Prevention of cardiovascular disease (CVD) and cancer (Li and Hu 2009); inflammatory, thrombotic and autoimmune disease (Calder 2006; Li and Hu 2009); coronary heart disease (Burr et al. 1989; De Lorgeril et al. 1994, 1996, 1999); hypertension (Appel et al. 1993, 1994); type 2 diabetes (Connor et al. 1993; Raheja et al. 1993), renal diseases (De Caterina et al. 1993; Donadio et al. 1994); rheumatoid arthritis (Kremer 1996); ulcerative colitis (Stenson et al. 1992); Crohn's disease (Belluzzi et al. 1996); chronic obstructive pulmonary disease (Shahar et al. 1994).

According to dietary recommendations of the American Heart Association Nutrition Committee (AHANC), it is suggested to consume fish at least two times per week or LC*ω*3-PUFA 500 mg/day to prevent and reduce the risk of heart disease (Krauss et al. 2000; Kris-Etherton et al. 2002; Kitessaa and Young 2011). The European Food Safety Authority (EFSA) panel recommends taking 250 mg n-3 LC-PUFA per day, in contrast to the Suggested Dietary Targets (SDT) in Australia for adult men and women that recommends 610 and 430 mg of EPA+DHA per day necessary to reduce the risk of CVD (Hu et al. 2002; Mozaffarian et al. 2005). Infant formulae should contain at least 0.2% of total fatty acids as DHA and 0.35% as AA (Kitessaa and Young 2011).

The conversion efficiency of ALA to EPA varies between 0.2% and 21%, and that of ALA to DHA varies between 0% and 9% (Andrew et al. 2006; Williams and Burdge 2006). The conversion of ALA to EPA and DHA is affected by multiple factors such as sex and competitive inhibition of Δ6-desaturase by LA and ALA. In adult men, the conversion efficiency of ALA to EPA is about 8% and EPA to DHA is too low (<0.1%), while the conversion efficiency to DHA in women is more than 9%, which may partly be the result of a lower proportion of ALA used for beta-oxidation in women compared to men (Andrew et al. 2006; Williams and Burdge 2006). The action of estrogen on Δ6-desaturase increases DHA concentration in pregnant and lactating women (Andrew et al. 2006; Williams and Burdge 2006).

## Importance of *ω*3/*ω*6 Ratio

Besides the amount of PUFA, the ratio of *ω*6/*ω*3 is known to be of nutritional importance as it is the key index for balanced synthesis of eicosanoids in the body (Steffens 1997). For optimal infant nutrition, the ratio of n-6/n-3 must be not higher than 10 (Gerster 1998). In Coastal states where mothers consumed high amounts of fish rich in n-3 PUFA, n-6/n-3 ratios were significantly lower than that of other countries (6.5 and 8.5, respectively) (Kneebone et al. 1985; Boersma et al. 1991). High consumption of plant oils rich in n-6 PUFA and consumption of relatively low marine foods (as source of n-3 PUFA) increases the n-6/n-3 ratio. When one has a diet rich in ALA and lower LA consumption levels, EPA and DHA in muscle tissue increased due to reduced competition for Δ6 desaturase. In most Indian consumers, the n-6/n-3 intake ratio is equal to 1/30-70, but the ideal ratio is 1/5-10 to protect human health. Japanese are the only people who take an ideal ratio of 1/2-4 and this is due to their consumption of seafood (Aleksandra et al. 2009). In communities in the west, consumption of *ω*6 is much higher than that of *ω*3; such that in the United States, consumption of *ω*6 is 10–30 times more than that of *ω*3. Nutritional scientists suggest the 2:1 to 4:1 n-6/n-3 ratio, which indicates a high consumption of seafood (Aleksandra et al. 2009).

## Long-Chain Polyunsaturated Fatty Acid Metabolism in Human Health

Linoleic and linolenic acids are synthesized in large quantities in plants, while they are not produced in humans and other mammals, so the fatty acids should be provided from external sources. Although humans can synthesize LC-PUFAs such as AA, DHA, and EPA by a series of desaturase and elongase enzymes from the precursor LA and ALA, the conversion efficiency is low in humans, so direct uptake appears to be significantly more effective (Horrocks and Yeo 1999). Mammals lack Δ12 and Δ15-desaturase activities, so they cannot synthesize LA and ALA from the precursor oleic acid (18:1Δ9) (Nakamura and Nara 2004). Synthesis of LC-PUFAs in humans and many other eukaryotes starts with Δ6 desaturation, which introduces a double bond between carbons 6 and 7 of LA and ALA, creating *γ*-linolenic and SDAs. These two fatty acids are then elongated by introducing two carbons via a Δ6-specific elongase to create DHGL and ETA. Subsequently, these fatty acids are desaturated to AA and EPA by a Δ5-desaturase (Qiu 2003). By Δ17-desaturase GLA, DHGLA, and AA can be converted to SDA, ETA, and EPA, respectively (Qiu 2003). In some algae and protists, another pathway is used, referred to as alternative Δ8-pathway. In this pathway, ALA is first elongated by a specific Δ9-elongase to eicosatrienoic acid (ERA; 20:3 Δ11, 14, 17), and then converted to ETA by Δ8-desaturase (Korn 1964; Wallis and Browse 1999; Sayanova et al. 2006; Zhou et al. 2007). Finally, in certain lower eukaryotes, in the Δ6-pathway, Δ4-desaturation of DPA produces DHA. In DHA-accumulating microbes, synthesis of DHA is a simple biochemical process where EPA is elongated by a specific Δ5-elongase to DPA, then converted to DHA by the action of a Δ4-desaturase. In the Sprecher pathway in mammals, EPA undergoes two rounds of elongation: first, generating DPA and then tetracosahexaenoic acid (THA; 24:5, n-3), which then yields 24:6 n-3 by Δ 6-desaturation. This C24 PUFA is then subjected to *β*-oxidation, by which it is chain-shortened by two carbons to yield the final product DHA. The pathway was identified by Howard Sprecher (Sprecher et al. 1999; Meyer et al. 2003; Venegas-Calerón et al. 2010).

## Various Sources of Long-Chain Polyunsaturated Fatty Acids

### Aquatic sources

Fish, especially oily fish of cold water such as code, tone, and mackerel, are excellent sources of long-chain n-3 polyunsaturated fatty acids (n-3 PUFA), predominantly EPA and DHA. Marine fish is a better source of *ω*-3 essential fatty acid (EFA), while freshwater fish is a good source of *ω*-6 EFA (Chukwuemeka et al. 2008; Pirestani et al. 2010; Nazemroaya et al. 2011). In the tissues of marine fish, the n-3/n-6 ratio on the average varied from 5 to 10, and in freshwater fish from 1 to 4 (Steffens 1997). Fatty acid compositions of different parts (head, tail, fins, and skin = HTFS, liver, viscera, and muscle tissue) of five commercially important fish species from the Persian Gulf (*Scomberomorus commersoni*, *Thunnus tonggol*, *Euthynnus affinis*, *Scomberomorus guttatus,* and *Dussumieria acuta*) as good sources of n-3 PUFA were studied. The richest sources of n-3 were HTFS in *S. guttatus* and *S. commersoni*, liver in *S. guttatus*, total body of *D. acuta*, liver of *E. affinis* and *T. tonggol*, followed by viscera of *E. affinis* (Sahari et al. 2013). Li and Hu (2009) investigated the n-3 PUFA content in common commercially available natural (wild) and cultured freshwater fishes. Four species of wild and cultured freshwater fishes — crucian carp, mandarin fish, silver fish, and snakeheaded fish — were studied. The fatty acids were analyzed and identified with capillary gas chromatography. Long-chain n-3 fatty acid contents in cultured samples were higher than in the wild, except for EPA, DPA, and DHA for mandarin fish; DHA for silver fish and ALA for snakeheaded fish were higher in the wild fish compared to the cultured fish. They concluded that it is not necessarily true that wild fishes are more nutritious (LC-3PUFA) than the cultured ones.

Main aquatic species that have been shown to have LC-3PUFA include fishes, shrimps, prawns, crabs, shellfishes, and algae. EPA and DHA content of aquatic species are shown in Table 1.

**Table 1:** Percent of EPA and DHA in fishs, crustaceans, and mollusks.

	EPA	DHA	Fish	EPA	DHA	References
Fish						
Anchovy	0.538	0.991	Haddock	0.076	0.162	Anonymous (2005), Andrew et al. (2006)
Bluefish	0.323	0.665	Halibut	0.091	0.374	Anonymous (2005), Andrew et al. (2006)
Turbot	0.09	0.123	Herring	0.97	1.179	Anonymous (2005), Andrew et al. (2006)
Carp	0.305	0.146	Lingcod	0.133	0.13	Anonymous (2005), Andrew et al. (2006)
Catfish	0.100	0.137	Mackerel, atlantic	0.504	0.699	Anonymous (2005), Andrew et al. (2006)
Caviar	2.741	3.800	Mullet	0.18	0.148	Anonymous (2005), Andrew et al. (2006)
Cod	0.004	0.154	Ocean perch	0.103	0.271	Anonymous (2005), Andrew et al. (2006)
Croaker	0.122	0.097	Pike (walleye)	0.11	0.288	Anonymous (2005), Andrew et al. (2006)
Dolphin fish	0.026	0.113	Pollock	0.091	0.451	Anonymous (2005), Andrew et al. (2006)
Grouper	0.035	0.213	Rock fish	0.181	0.262	Anonymous (2005), Andrew et al. (2006)
Sablefish	0.867	0.92	Smelt	0.353	0.536	Anonymous (2005), Andrew et al. (2006)
Salmon (atlantic), farmed	0.69	1.457	Spot	0.282	0.526	Anonymous (2005), Andrew et al. (2006)
Sardine	0.473	0.509	Sturgeon	0.249	0.119	Anonymous (2005), Andrew et al. (2006)
Shad	1.086	1.321	Sucker	0.244	0.371	Anonymous (2005), Andrew et al. (2006)
Shark	0.316	0.527	Swordfish	0.138	0.681	Anonymous (2005), Andrew et al. (2006)
Sheepshead	0.083	0.107	Tilefish	0.172	0.733	Anonymous (2005), Andrew et al. (2006)
Snapper	0.048	0.273	Trout	0.259	0.677	Anonymous (2005), Andrew et al. (2006)
Tuna, light canned in oil	0.027	0.101	Whitefish	0.406	1.206	Anonymous (2005), Andrew et al. (2006)
Tuna, light canned in water	0.047	0.223	Whiting	0.283	0.235	Anonymous (2005), Andrew et al. (2006)
Wolffish	0.393	0.405	Common kilka	5.67	9.93	Anonymous (2005), Pirestani et al. (2010)
Caspian kutum	4.55	7.14	Golden gray mullet	7.56	3.86	Anonymous (2005), Andrew et al. (2006)
Mollusks						
Mollusk, clam	0.138	0.146	Mollusk, cuttlefish	0.078	0.132	Anonymous (2005), Andrew et al. (2006)
Mollusk, mussel, blue	0.276	0.506	Mollusk, octopus	0.151	0.162	Anonymous (2005), Andrew et al. (2006)
Mollusk, oyster	0.229	0.211	Mollusk, oyster, pacific	0.876	0.500	Anonymous (2005), Andrew et al. (2006)
Mollusk, Conch	0.061	0.067	Mollusk Scallop	0.071	0.101	Anonymous (2005), Andrew et al. (2006)
Crustaceans						
Spiny lobster	0.3	0.1	Shrimp	0.3	0.2	Gonzάlez-Félix et al. (2003), Anonymous (2005)
Mussles	0.2	0.3	Crab, Alaska king	0.2	0.2	Gonzάlez-Félix et al. (2003), Anonymous (2005)

### Microbial polyunsaturated fatty acid

Microorganisms including bacteria, fungi, algae, mosses, and protozoa can synthesize a variety of PUFAs (Ratledge and Wilkinson 1988). The bacterium *Moritella marina*, the fungi *Thraustochytrium* spp. and *Entomophthora* spp., and some species of the class *Thraustochytriales* including *Thraustochytrium aureum*, *Thraustochytrium roseum*, and *Thraustochytrium* sp. ATCC 20892 are microorganisms that produce high levels of DHA (Wu et al. 2005; Perveen et al. 2006). The membranes of *Vibrio marinus* strain MP-1 contain substantial amounts of DHA and usually the genes for DHA synthesis are derived from it, while *Shewanella marinintestina* contains a significant amount of EPA (Morita et al. 1999; Allen and Bartlett 2002). Some microorganisms that produce polyunsaturated fatty acid are considered in Table 2.

**Table 2:** Selected microorganisms for polyunsaturated fatty acid production.

PUFA	Strain	Reference
AA	**Bacteria, fungi**	
	Mortierella alpine	Horrobin (1995)
	Conidiobolus nanodes	Ratledge and Wilkinson (1988)
	Entomophthora exitalis	Leman (1997)
	Blastocladiella emersonit	Sessler and Ntambi (1998)
	**Microalgae**	
	Chrysophyceae *Monochrysis lutheri*	Yongmanitchai and Ward (1989)
	*Pseudopedinella* sp.	Yongmanitchai and Ward (1989)
	*Coccolithus huxleyi*.	Yongmanitchai and Ward (1989)
	*Cricosphaera carterae*	Yongmanitchai and Ward (1989)
	*C. elongata*	Yongmanitchai and Ward (1989)
	Eustigmatophyceae	
	*Monodus subterranneus*	Qiang et al. (1997)
	*N. salina*	Yongmanitchai and Ward (1989)
	Prasinophyceae (*Hetermastrix rotundra)*	Yongmanitchai and Ward (1989)
	Chlorophyceae	
	*Chlorella minutissima*	Seto et al. (1984)
	*Parietochloris incisa*	Bigogno et al. (2002)
	Cryptophyceae (*Cryptomonas maculate*)	Yongmanitchai and Ward (1989)
	Bacillariophyceae (*Thalassiosiosira pseudonana)*	Cobelas and Lechado (1989)
	Dinophyceae (*Amphidinium carteri*)	Cobelas and Lechado (1989)
	Phaeophyceae	
	*Desmarestia acculeata*	Pohl and Zurheide (1979)
	*Dictyopteris membranacea*	Hofman and Eichenberger (1997)
	*Ectocarpus fasciculatus*	Makewicz et al. (1997)
	Prasinophyceae (*Ochromonas danica*)	Vogel and Eichenberger (1992)
	Rhodophyceae	
	*Gracilaria confervoides*	Pohl and Zurheide (1979)
	*Phycodrys sinuosa*	Pohl and Zurheide (1979)
	*Porphyridium cruenturn* 1380-1a	Cohen (1990)
	**Diatom**	
	*Asterionella japonica*	Yongmanitchai and Ward (1989)
	*Amphora coffeaformis*	Renaud et al. (1999)
	*Chaetoceros* sp.	Renaud et al. (1999)
	*Fragilaria pinnara*	Renaud et al. (1999)
	*Navicula saprophila*	Kitano et al. (1997)
	*Nitzschia laevis*	Wen and Chen (2000)
EPA	**Bacteria, fungi**	
	Mortierella alpine	Horrobin (1995)
	Mortierella elongate	Radwan (1991)
	Pythium ultimum	Shimizu and Jareonkitmongkol (1995)
	Shewanella putrefaciens	Eroshin et al. (1996)
	**Microalgae**	
	Chrysophyceae	
	*(Monochrysis lutheri*	Yongmanitchai and Ward (1989)
	*Pseudopedinella* sp.	Yongmanitchai and Ward (1989)
	*Coccolithus huxleyi*	Yongmanitchai and Ward (1989)
	*Cricosphaera carterae*	Yongmanitchai and Ward (1989)
	*C. elongata*	Yongmanitchai and Ward (1989)
	*Isochrysis galbana)*	Molina Grima et al. (1992)
	Eustigmatophyceae	
	*Monodus subterranneus*	Qiang et al. (1997)
	*Nannochloropsis* sp.	Sukenik (1991)
	*Nannochloris* sp.	Yongmanitchai and Ward (1989)
	*N. salina)*	Yongmanitchai and Ward (1989)
	Prasinophyceae	
	*Hetermastrix rotundra*	Yongmanitchai and Ward (1989)
	Chlorophyceae	
	*Chlorella minutissima*	Seto et al. (1984)
	*Parietochloris incisa*	Bigogno et al. (2002)
	Cryptophyceae	
	*Chromonas* sp.	Renaud et al. (1999)
	*Cryptomonas maculate*	Yongmanitchai and Ward (1989)
	*Cryptomonas* sp.	Yongmanitchai and Ward (1989)
	*Rhodomonas* sp.	Renaud et al. (1999)
	Bacillariophyceae	
	*Thalassiosiosira pseudonana*	Bigogno et al. (2002)
	Phaeodactylum tricornutum	Guedes et al. (2011)
	Phaeophyceae	
	*Desmarestia acculeata*	Pohl and Zurheide (1979)
	*Dictyopteris membranacea*	Hofman and Eichenberger (1997)
	*Ectocarpus fasciculatus*	Pohl and Zurheide (1979)
	Rhodophyceae	
	*Phycodrys sinuosa,*	Pohl and Zurheide (1979)
	*Porphyridium cruenturn* 1380-1a;	Cohen (1990)
	Prymnesiophyceae (*Pavlova luteri*)	Guedes et al. (2011)
	**Diatom**	
	*Asterionella japonica*	Yongmanitchai and Ward (1989)
	*Amphora coffeaformis*	Renaud et al. (1999)
	*Biddulphia sinensis*	Yongmanitchai and Ward (1989)
	*Chaetoceros* sp.	Renaud et al. (1999)
	*Fragilaria pinnara*	Renaud et al. (1999)
	*Navicula incerta*	Tan and Johns (1996)
	*Navicula pelliculosa*	Tan and Johns (1996)
	*Navicula saprophila*	Kitano et al. (1997)
	*Nitzschia closterium*	Renaud et al. (1994)
	*Nitzschia frustulum*	Renaud et al. (1994)
	*Nitzschia laevis*	Wen and Chen (2000)
	*Phaeodactylum tricornutum*	Yongmanitchai and Ward (1991)
	*Skeletonema costatum*	Blanchemain and Grizeau (1999)
DHA	**Bacteria, fungi**	
	Schyzotrichium SR21, Crypthecodinium cohnii	Ratledge (1989)
	Schyzochytrium aggregatum, Thraustochytrium roseurn	
	Thraustochytrium aureum, Vibrio spp., Rhodopseudomonas spp.	
	**Microalgae**	
	Chrysophyceae (*Isochrysis galbana)*	Molina Grima et al. (1992)
	Prasinophyceae (*Hetermastrix rotundra)*	Yongmanitchai and Ward (1989)
	Cryptophyceae	
	*Chromonas* sp.	Renaud et al. (1999)
	*Cryptomonas* sp.	Yongmanitchai and Ward (1989)
	*Rhodomonas* sp.	Renaud et al. (1999)
	Dinophyceae (*Amphidinium carteri*)	Cobelas and Lechado (1989)
	Prymnesiophyceae (*Pavlova luteri*)	Guedes et al. (2011)
	**Diatom**	
	*Asterionella japonica*	Yongmanitchai and Ward (1989)
	*Amphora coffeaformis*	Renaud et al. (1999)
	*Biddulphia sinensis*	Yongmanitchai and Ward (1989)
	*Chaetoceros* sp.	Renaud et al. (1999)
	*Cylindrotheca fusiformis*	Tan and Johns (1996)
	*Fragilaria pinnara*	Renaud et al. (1999)

Stearic acid is converted to oleic acid by the addition of the first double bond to its Δ9 position; then desaturated by Δ12 desaturase to yield LA, which may be consequently converted by Δl5 desaturase to ALA. Thus, oleic acid, LA, and ALA are basic precursors of *ω*9, *ω*6, and *ω*3 fatty acids. The next steps are desaturation of fatty acid precursors by Δ6 desaturase, followed by elongations and subsequent desaturation(s) to produce the C20 and C22 PUFAs, respectively. The *ω*9 family of PUFA is synthesized from oleic acid and sequential participation of Δ6 desaturase, elongase, and Δ5 desaturase to finally produce MA. Linoleic acid via Δ6 desaturase and Δ6 elongase steps was generated to GLA and DHGL, then by Δ5 desaturase, Δ5 elongase was produced to AA and adrenic acid (22:4 cis 7, 10, 13, 16) and the final step was done by Δ5 desaturase to produce *ω*6 DPA. A similar pathway with the same enzymes can be found in *ω*3 family which can produce EPA and DHA. There are two biosynthetic pathways for the production of *ω*3 PUFAs in microorganisms (Certik et al. 1998). In the first biosynthesis pathway, which is temperature independent, EPA, DPA *ω*3, and DHA are produced via desaturation and elongation of ALA. The second pathway is temperature dependent and includes the conversion of *ω*6 fatty acids to *ω*3 PUFAs synthesized by two possible enzymes, Δ15 and Δ17 desaturases (Certik and Shimizu 1999).

#### Microalgae

Microalgae are microorganisms that obtain energy from light and can produce valuable metabolites, for example, antimicrobials, antioxidants, and PUFAs (Certik and Shimizu 1999; Guedes et al. 2011).

For further details on marine microalgal synthesis of PUFAs, see Tonon et al. (2004) and Guedes et al. (2011). This synthetic route also seems to occur in cyanobacteria, although the specific enzymatic details may differ. The most common pathway for LC-PUFA synthesis is the Δ6 desaturase/Δ6 elongase. LA and ALA are first desaturated by a Δ6 desaturase via the addition of a double bond by a Δ6 desaturase to form GLA and SDA; elongated to form DHGLA and ETA, respectively; then another double bond is added by a Δ5 desaturase to form AA, EPA; elongated to form *ω*3-DPA; and finally, a double bond is added to form DHA. A less common pathway is Δ9 elongation/Δ8 desaturation, by which LA or ALA is first elongated by a Δ9 elongase to produce eicosadienoic acid (20:2 Δ11, 14) (EDA) or ERA (20:3 Δ11, 14, 17), subsequently desaturated by a Δ8 desaturase to yield DHGLA or ETA, respectively. However, the formation of DHA from EPA is known to occur via two distinct mechanisms in eukaryotes; in lower eukaryotes (as in the case of microalgae), EPA is elongated to *ω*3-DPA, and a double bond is then directly introduced thereto by a Δ4 desaturase to yield DHA. To synthesize long-chain *ω*6 PUFA (e.g., *ω*6-DPA from LA), the same alternating desaturation and elongation steps via either the Δ4 desaturase route or the *β*-oxidation route are followed (Zhou et al. 2007).

#### Diatoms

Diatoms are thought of as a high-quality food source (D'Ippolito et al. 2002). Diatoms are abundant in most aquatic habitats and are considered to be the most important primary producers in marine food chains (D'Ippolito et al. 2004). In Table 2, the amount and name of diatoms that produce LC-3PUFA are presented.

### Animal sources

Fatty acids found in animal sources such as meat of beef, lamb, pork, and poultry; milk and dairy products; and eggs under standard production systems are summarized in Table 3. These will be influenced by the diet composition, the digestive system of the animal, and the biosynthetic processes within the animal.

**Table 3:** Principle fatty acids in milk, beef, pork, lamb, chicken, and eggs (g/100 g total FA).

Fatty acid	18:2 n-6	18: 3n-3	20:4 n-6	20:5 n-3	22:5 n-3	22:6n-3	References
Game meat							
Skeletal muscle	16.49	2.97	8.31	1.19	1.99	0.77	Boyd Eaton et al. (1998)
Brain	0.35	0.04	4.4	0.17	25.0	8.05	Boyd Eaton et al. (1998)
Liver	10.19	1.61	10.45	1.45	3.42	3.98	Boyd Eaton et al. (1998)
Bone marrow	7.08	86.0	16.0	0.08	0.06	0.07	Boyd Eaton et al. (1998)
Separable fat	12.33	32.82	0.64	0.6	0.15	0.26	Boyd Eaton et al. (1998)
Beef muscle	2.8	0.8	0.5	0.3	0.5	ND	Woods and Fearon (2009)
Beef fat	1.0	0.5	ND	ND	ND	ND	Woods and Fearon (2009)
Lamb muscle	1.8	1.2	0.5	0.3	0.4	0.1	Woods and Fearon (2009)
Lamb fat	1.2	1.1	0.1	Tr	0.1	ND	Woods and Fearon (2009)
Pork muscle	14.8	1.4	1.1	0.3	0.5	0.3	Woods and Fearon (2009)
Pork fat	14.8	1.5	0.2	ND	0.2	0.2	Woods and Fearon (2009)
Chicken (dark meat)	16.6	2.6	0.4	ND	0.4	0.4	Woods and Fearon (2009)
Chicken (light meat)	13.7	1.7	0.8	Tr	0.8	0.8	Woods and Fearon (2009)
Milk	1.9	0.5	ND	ND	Tr	ND	Woods and Fearon (2009)
Eggs	17.2	0.9	ND	ND	ND	ND	Woods and Fearon (2009)

#### Meat sources

Both muscle and adipose tissues of meat contain ALA and the long-chain n-3 PUFA, including EPA, DPA, and DHA, which play an important role in human diet. Unlike fish, red meat is the main dietary source of DPA, which accumulates in mammalian muscle (Givens and Gibbs 2006). DPA reduces the risk of atherosclerotic and acute coronary events in middle-aged men and has significant health benefits compared to EPA and DHA in reducing the risk of CVD (Rissanen et al. 2000; Hino et al. 2004; Howe et al. 2006). The fatty acid composition of meat will vary by animal's age, sex, breed, diet, and within the cut of meat (Wood and Enser 1997). There is an interest in modifying its nutritional value. Fatty acid composition of the monogastric animals is a reflection of the dietary fatty acids, while in ruminants, the biohydrogenation in the intestine of rumen (i.e., saturation of the dietary unsaturated fatty acids) is responsible for smaller variations in intramuscular fatty acid composition (Wood et al. 1999). In red meat such as beef, lamb, and pork and white meat including poultry, the LC-PUFA (*ω*3 and *ω*6) can also be produced from their dietary precursors, ALA and LA. Phospholipids in muscle are identified by a high PUFA content (20–50% of the total fatty acids in the phospholipids), mainly containing long-chain fatty acids with 18, 20, and 22 carbons and two to six double bonds. Phospholipid composition is less affected by diet. Therefore, the PUFA proportion of the phospholipids is absolutely controlled by a complex enzymatic system, including desaturases and elongases, which are responsible for the conversion of both the precursors LA and ALA to AA, EPA, DPA, and DHA (Raes et al. 2004). In contrast to phospholipids, the content of triacylglycerols varies widely, between 0.2 and 5 g/100 g of fresh tissue (Gandemer 1999). The fatty acid content of triacylglycerol, which consists of saturated fatty acids (SFA) and monounsaturated fatty acids (MUFA), is more than that of PUFA, since the PUFA (predominantly LA and ALA) content in triacylglycerols may vary between 2 and 30 g/100 g of the total fatty acids. The PUFA content in the triacylglycerols is mainly influenced by species (2–3% and 7–15% PUFA in the triacylglycerols of beef and pork, respectively; Gandemer 1999; Raes et al. 2004).

The LCn-3 PUFA (EPA and DHA) amount in beef and lamb is lower than that in oily fish (0.28 and 0.52 vs. 19.9 mg/g) (Irie and Sakimoto 1992; Morgan et al. 1992; Ishida et al. 1996; Leskanich et al. 1997), marine algae (Fredriksson et al. 2006; Sardi et al. 2006), or dietary sources rich in *α*-linolenic acid such as linseed (flaxseed) (Cunnane et al. 1990; Cherian and Sim 1995; Romans et al. 1995; Ahn et al. 1996), perilla (Siriamornpun et al. 2006), echium oil (Berti et al. 2007), chia (Ayerza and Coates 2000), and canola oil (from rapeseed) (Rhee et al. 1988) which induce to increase the EPA, DPA, DHA, and linolenic acid contents to 100%, 29%, 35%, and 55%, respectively. However, the linoleic acid, AA, and 22:4 contents decrease to 14%, 16%, and 35%, respectively, in lamb, beef, and pork; and in monogastric animals such as ostriches. An increase in n-3 LCPUFA in pork diet may reduce the shelf life of the product, with off-odors and flavors, and induce impairment of meat color due to oxidation of the PUFA (Enser et al. 2000; Wood et al. 2003).

#### Milk sources

Milk and dairy products play an important role in human nutrition. In contrast to cow milk and infant formula, human milk contains small but significant amounts of LC-PUFA, particularly AA and DHA that are necessary for optimal development of the brain (Crawford et al. 1981), the retina (Neuriger et al. 1984), and other infant tissues. Total PUFA in cow milk is very low, while in infant formula and in human milk, the percentage of PUFA is significant. As can be seen in Table 4, the linoleic acid content of human milk and baby formula was similar, and higher than that in cow milk. However, the linolenic acid and total n-3 content were slightly lower in human milk and infant formula when compared with cow milk, but LC-PUFAs such as AA, EPA, DPA, and DHA were detected exclusively in human milk (Table 4). The n-6/n-3 ratio in human milk was significantly higher than those for infant formula and cow milk. DHA and AA contents in human milk vary between 0.06–1.4% and 0.24–1%, respectively. In addition, DHA and other LC-3PUFA contents increase in women living in coastal areas (Kitessaa and Young 2011). Dietary sources rich in *α*-linolenic acid such as echium oil (Kitessaa and Young 2011) and fish/soybean oil (Kitessa et al. 2003) can improve the profile composition of LC-3PUFA except DHA in animal milk such as goat, sheep, cow, etc. (Aleksandra et al. 2009).

**Table 4:** The percentages of polyunsaturated fatty acids (PUFA) in cow's milk, baby formula, and human milk.

Fatty acid	Cow's milk	Baby formula	Human milk	References
18:2 n-6	1.02 ± 0.01	14.03 ± 1.00	16.29 ± 3.63	Aleksandra et al. (2009)
18:3 n-6	ND	ND	0.12 ± 0.10	Aleksandra et al. (2009)
18:3 n-3	2.92 ± 0.12	0.80 ± 0.20	0.49 ± 0.23	Aleksandra et al. (2009)
20:2 n-6	ND	ND	0.56 ± 0.13	Aleksandra et al. (2009)
20:3 n-6	ND	ND	0.57 ± 0.18	Aleksandra et al. (2009)
20:4 n-6	ND	ND	0.51 ± 0.11	Aleksandra et al. (2009)
20:5 n-6	ND	ND	0.10 ± 0.05	Aleksandra et al. (2009)
22:4 n-6	ND	ND	0.19 ± 0.13	Aleksandra et al. (2009)
22:5 n-3	ND	ND	0.09 ± 0.04	Aleksandra et al. (2009)
22:6 n-3	ND	ND	0.19 ± 0.07	Aleksandra et al. (2009)
Total PUFA	3.60 ± 0.57	15.00 ± 2.00	19.10 ± 3.91	Aleksandra et al. (2009)
n-6	1.02 ± 0.10	14.06 ± 1.00	18.24 ± 3.80	Aleksandra et al. (2009)
n-3	2.92 ± 0.12	0.80 ± 0.10	0.86 ± 0.29	Aleksandra et al. (2009)
n-6/n-3	0.35 ± 0.02	17.42 ± 0.80	22.65 ± 6.16	Aleksandra et al. (2009)

#### Egg yolk

Egg yolk is a good source of PUFAs, especially DHA. Egg yolk contains 0.1% EPA, 0.7% DHA, and 0.8% ALA. Attempts have been made to produce eggs rich in EPA or DHA for people not consuming fish products. The EPA and DHA contents of eggs can be increased by feeding chicken with diets containing fish oil. Nevertheless, this negatively influences the palatability (flavor, taste, and odor) of the eggs (Huang et al. 1990). Another possibility to raise the EPA and DHA contents is through feeding the chicken by a diet rich in ALA (Jiang et al. 1991) such as soybean and linseed (Beynen 2002). ALA can be converted to EPA and DHA by the desaturase and elongase in chicken liver and the LC-3PUFA are synthesized. The conversion of ALA into EPA and DHA may be inhibited by high LA intakes (James et al. 2000). Simopoulos and Salem (1992) compared the fatty acid content of egg yolks from hens fed four different diets as a source of DHA including Greek eggs (Greek eggs came from the Ampelistra farm in Greece), supermarket eggs, fish-meal eggs, and flax eggs that are good sources of *ω*3 PUFA.

In PUFA enrichment of the Belovo PUFA-enriched egg, *ω*3 PUFA content is increased and on the other hand the *ω*-6 PUFA content is decreased slightly. The *ω*3 PUFA fraction in the Belovo egg is increased about 20-fold in ALA content and approximately twofold in long-chain *ω*3 PUFAs (i.e., sum of EPA, DPA, and DHA). ALA comprises about 80% of the *ω*3 PUFA content of the Belovo PUFA-enriched egg. The Belovo PUFA-enriched egg has a 1:1 *ω*3/*ω*6 PUFA ratio, while fish oils typically have a *ω*3/*ω*6 PUFA ratio of greater than 6:1. Furthermore, the doses of *ω*3 LC-PUFA in fish oil supplements are 10-fold higher than 0.1 g of *ω*3 LC-PUFA provided by one Belovo egg, which is the daily consumption recommendation. The Belovo PUFA-enriched egg is currently being marketed under the name Christopher® egg (Belovo Inc., Pinehurst, NC). According to the reports, these eggs have a PUFA composition consisting of equal amounts of *ω*3 and *ω*6 PUFAs (i.e., 1:1 balanced ratio) (Steele 2006); total *ω*3 PUFA content of 660 mg per 50 g egg (Steele 2006), and *ω*3 PUFA content distributed as 83% ALA (550 mg/50 g egg) and 17% *ω*3 LC-PUFA (110 mg/50 g egg). The relative proportion of the *ω*3 LC-PUFA in the enriched egg is 1:2:8 for EPA, DPA, and DHA, respectively (Steele 2006).

## Plant Polyunsaturated Fatty Acid Production

### Production of *γ*-linolenic acid and SDA, EPA, AA, and DHA in transgenic plants

Both GLA and SDA are naturally produced in certain plants such as borage, primrose seed, hemp, and black current seed oils. These plants are difficult to cultivate and extraction of the oils is performed with relatively poor efficiency. Thus, there is considerable interest in producing GLA and SDA in oilseed crops. Production of GLA or SDA in oilseeds that contain significant amounts of LA or ALA requires only the expression of a Δ6-desaturase gene. The 6-desaturases gene was isolated from the fungus *Mortierella alpina* (Huang et al. 1999; Sakuradani et al. 1999), cyanobacteria (i.e., cyanobacterium *Synechocystis*) (Reddy et al. 1993; Sayanova et al. 2006), borage (Reddy et al. 1993), nematode (i.e., *Caenorhabditis elegans*) (Napier et al. 1998), mammals (Aki et al. 1999; Cho et al. 1999; Sato et al. 2001), and *Physcomitrella patens* (Girke et al. 1998). In this regard, expression of a Δ6-desaturase gene has been done to induce accumulation of 0.1–2% SDA and GLA of seed fatty acids in flax seed (Qiu et al. 2002), 10% GLA and 3% SDA in *Brassica juncea* (rapeseed) (Hong et al. 2002), and significant amounts of GLA (13.2%) in *Brassica napus* (Hong et al. 2002), and soybean (*Glycine max*) (Sato et al. 2004). In tobacco (*Nicotiana tabacum*), GLA and SDA represent only about 1 and 1–3% of C18 fatty acid content (Reddy and Thomas 1996).

The genes encoding Δ6-desaturases, Δ6-elongase components, and Δ5-desaturases have been cloned from a variety of organisms, including higher plants, algae, mosses, fungi, nematodes, and humans. These genes are required for DHA production (Qi et al. 2004). The first step in the production of AA or EPA from GLA or SDA is performed by elongases that are capable of elongating SDA and GLA to DHGLA and ETA. This is followed by the pathway that requires the activity of a Δ5-desaturase to produce AA or EPA. Meyer et al. (2003) explained isolation and characterization of elongases acting on EPA from a number of species including the fish *Oncorhynchus mykiss*, the frog *Xenopus laevis*, the sea squirt *Ciona intestinalis*, and the algae *Ostreococcus tauri* and *Thalassiosira pseudonana*. These enzymes can produce DHA (Leonarda et al. 2004). Vrinten et al. (2007) reported the use of the same *M. alpina* Δ5- and Δ-6 desaturases and *M. alpina* elongase achieving EPA levels of 9.3% in somatic soybean embryos. The Δ5-desaturase appeared to be quite efficient, as it produced up to 2% AA in tobacco, and accumulated up to 1.5% AA and 1% EPA in flax. The Δ4-desaturase gene capable of acting on fatty acid *ω*3 DPA was expressed in *B. juncea* plants with exogenous DPA, which led to an accumulation of 3-6% DHA in leaf tissue. In another study, by expression of Δ5- and Δ6-desaturases and a Δ6-elongase from *M. alpina*, plus a *Pavlova* sp. Δ5-elongase, a *Schizochytrium aggregatum* Δ4-desaturase, and a *S. diclina* Δ17-desaturase in transgenic soybean somatic embryos, an accumulation of up to 3.3% DHA of total fatty acid was induced (Vrinten et al. 2007). Qi et al. (2004) reported production of two very long-chain PUFAs — AA and EPA — in significant quantities in a higher plant. Genes encoding a Δ9-specific elongase from *Isochrysis galbana*, a Δ8-desaturase from *Euglena gracilis*, and a Δ5-desaturase from *Mortierella alpine* were transferred to *Arabidopsis thaliana* and led to production of significant amounts of EPA and AA in this plant. Wu et al. (2005) investigated transfer of a Δ5-desaturase from *Thraustochytrium* sp., a Δ6-desaturase from *P. irregulare*, and a Δ6-elongase from *P. patens* to *B. juncea*. The Δ6-elongase led to production of GLA, with high conversion efficiency of the Δ5-desaturase, resulting in the production of 7.3% AA and 0.8% EPA.

### Plant resource of AA, EPA, DPA, and DHA

Unexpectedly, Guil et al. (1996) identified LC-PUFAs in some plants. A total of 20 wild vegetables were identified. Plant materials were harvested, washed with water, dried, and powdered. The total lipid content of the plant was extracted in a Soxhlet apparatus with light petroleum. The oil content was analyzed by GC. The LC-PUFA is shown in Table 5.

**Table 5:** Fatty acid percentage contents in edible wild plants.

Species	18:3 *ω*3 ALA	18:4 *ω*3 SDA	18:3 *ω*6 GLA	20:5*ω*3 EPA	20:4*ω*6 AA	22:5*ω*3 DPA	22:6*ω*3 DHA	References
*Amaranthus viridis* L. (amaranth)	24.34	0.00	0.25	0.93	0.00	0.00	0.00	Guil et al. (1996)
*Beta maritima* L. (wild beet)	29.44	0.50	0.17	0.54	0.52	0.49	0.65	Guil et al. (1996)
*Cakile maritima Scopoli* (sea rocket)	23.84	0.38	0.00	0.09	0.00	0.00	0.00	Guil et al. (1996)
*Cardaria draba* L. (hoary cress)	30.56	0.62	0.60	2.16	0.56	0.00	0.00	Guil et al. (1996)
*Chenopodium album* L. (goosefoot)	44.82	0.17	0.00	0.36	1.30	0.00	0.00	Guil et al. (1996)
*Chenopodium murale* L. (goosefoot)	36.04	0.14	0.00	0.41	1.01	0.00	0.00	Guil et al. (1996)
*Chenopodium opulifolium Schrader* (goosefoot)	33.02	0.00	0.57	3.06	0.00	0.74	2.30	Guil et al. (1996)
*Crithmum maritimum* L. (rock samphire)	9.98	0.42	0.25	0.76	0.00	0.76	0.00	Guil et al. (1996)
*Malva sylvestris* L. (common mallow)	42.22	0.29	0.50	0.00	5.30	0.00	0.00	Guil et al. (1996)
*Parietaria diffusa Mert*. (pelitory-of-the-wall)	21.18	3.64	1.99	0.00	0.00	0.00	0.00	Guil et al. (1996)
*Pichris echioides* L. (ox-tongue)	43.20	0.20	0.42	0.00	0.00	0.15	0.00	Guil et al. (1996)
*Plantago major* L. (plantain)	33.32	2.02	0.00	1.27	1.02	0.00	1.47	Guil et al. (1996)
*Portulaca oleracea* L. (purslane)	32.60	0.00	0.27	0.00	0.00	0.00	0.00	Guil et al. (1996)
*Rumex crispus* L. (curly dock)	41.21	1.73	0.00	0.12	0.00	0.00	0.00	Guil et al. (1996)
*Salicornia europaea* L. (glasswort)	28.03	0.44	0.55	0.39	0.00	0.00	0.00	Guil et al. (1996)
*Sisymbrium irio* L. (hedge mustard)	31.04	0.19	0.00	0.55	0.32	0.21	0.83	Guil et al. (1996)
*Sonchus oleraceus* L. (sow-thistle)	43.58	0.09	0.34	0.35	0.00	0.25	0.00	Guil et al. (1996)
*Sonchus tenerrimus* L. (sow-thistle-of-the-wall)	30.33	0.11	0.35	0.00	1.83	0.38	0.00	Guil et al. (1996)
*Stellaria media Villars* (chickweed)	22.75	4.68	2.40	0.42	0.41	0.00	0.00	Guil et al. (1996)
*Verbena offieinalis* L. (vervain)	54.99	0.00	0.25	0.60	0.62	0.00	0.00	Guil et al. (1996)

### Plant resource of *α*-linoleic acid (18:3n3)

ALA (18:3n-3) is an 18-carbon fatty acid with three double bonds at carbons 9, 12, and 15. It is an essential n-3 fatty acid which is a required nutrient for humans and can be obtained through diets including both plant and animal sources. ALA can be converted by elongases and desaturases to other beneficial n-3 fatty acids such as EPA and DHA, which contribute to normal brain development, normal vision, and decreased risk of CVDs. Animals and mammals cannot provide ALA, while plants are the richest source for supplying it. This section summarized fruit, spice, herb, and seed oils rich in ALA (Table 6).

**Table 6:** Fatty Acid Compositions (g Fatty Acid/100-g Oil) of Seed Oils fruits, herbs and seeds with Relatively High concentrations of *α*-Linolenic Acid (18:3 n-3).

Fatty acid	Black Raspberry	Red Raspberry	Boysenberry	Marionberry	Blueberry	Cranberry	Buckthorn sinensis	Buckthorn Rhamnoides	O.Basilicum (basil)	Hemp
16:0	1.2–1.6	1.2–2.7	4.2	3.3	5.7	3.0–7.8	7.7–9.6	6.7–8.2	6.8–8.8	5.8–6.7
18:0	Trace	1.0	4.5	3.1	2.8	0.2–1.9	2.1–3.3	2.3–4.1	2.0–2.8	2.6–3.2
18:1	6.2–7.7	12.0–12.4	17.9	15.1	22.8	20.0–27.8	12.9-26.1	13.7–20.0	8.7–11.6	9.9–15.6
18:2	55.9–57.9	53.0–54.5	53.8	62.8	43.5	35.0–44.3	38.2-43.6	36.7–43.0	18.3–21.7	53.4–60.0
18:3 n-3	35.2–35.3	29.1–32.4	19.5	15.7	25.1	22.3–35.0	20.2-36.3	25.4-36.0	57.4–62.5	15.1–19.4
others	ND	ND	ND	ND	ND	2.5	1.9–2.5	1.8–3.8	trace	0–1.8
	Yu et al. (2005)	Yu et al. (2005)	Yu et al. (2005)	Yu et al. (2005)	Yu et al. (2005)	Yu et al. (2005)	Yu et al. (2005)	Yu et al. (2005)	Yu et al. 2005;^117^	Yu et al. (2005), Angers et al. (1996)

Fatty acid	Perilla	Camelina	Chia	Flax seed	Malaysian rubber seed oil	Rose fruit	Walnut	Lupin	Canola	Soybean
16:0	7	6	6	5.3	8.56	1.7–3.1	8.1	8.7	3.8	11.2
18:0	2	2	3	3.3	10.56	1.7–2.5	2.8	0.8	1.7	4.1
18:1	14	13	7	17.9	22.95	14.7–18.4	15.9	45.8	58.2	24.3
18:2n-6	17	16	20	17.7	32.28	48.6–54.4	59.7	24.1	20.1	54.6
18:3n-3	61	39	63	58.7	19.22	16.4–18.4	13.1	11.3	9.6	8.3
	Gunstone et al. (1994), Siriamornpun et al. (2006)	Yu et al. (2005), Budin et al. (1995)	Coates and Ayerza (1998), Yu et al. (2005)	Gunstone et al. (1994), Hassan-Zadeh et al. (2008)	Salimon and Abdullah (2009)	Yu et al. (2005)	Venkatachalam and Sathe (2006)	Bhardwaj et al. (2004)	Przybylski et al. (2005)	Yu et al. (2005)

#### Black raspberry seed oil (*Rubus occidentalis* L., cv. Jewel)

Black raspberry is a member of the genus *Rubus* from the Rosaceae family, which is also known as caneberries. The concentration of *α*-linolenic acid is 35% of the total fats, and unsaturated fatty acids comprise 98–99% of the content. LA is the predominant fatty acid but ratios of n-6 to n-3 fatty acids are very low at 1.6:1 (Table 6). The overall fatty acid composition of black raspberry seed oil is very similar to red raspberry seed oil (Parry and Yu 2004).

#### Red raspberry seed oil (*Rubus ideaus*)

Red raspberry seed oil contains 29.1–32.4% ALA. The amounts of both LA and ALA are lower than those in black raspberry seed oil and the ratios of n-6 to n-3 fatty acids are 1.6–1.8:1 (Table 6) (Yu et al. 2005).

#### Boysenberry seed oil (*Rubus hybrid*)

Boysenberry seed oil is similar to the oil of other caneberries (black raspberry, red raspberry, and marionberry), having a high percentage (19.5%) of ALA and a ratio of n-6 to n-3 fatty acids of 2.8:1.The total unsaturated fatty acids comprise over 91% of the seed oil and PUFAs are very high at 73.3%, but stearic, palmitic, and total SFA are higher than all the other caneberry seed oils (Table 6) (Yu et al. 2005).

#### Marionberry (*Rubus hybrid*) seed oil

Marionberry is a blackberry hybrid and another member of the caneberry family. The oil was shown to contain a relatively high percentage of n-3 fatty acids in the form of ALA (15.7%). This amount is lower than that of the other caneberry seed oils, such as black raspberry, red raspberry, and boysenberry seed oils, tested under the same conditions. The n-6 to n-3 fatty acid ratio is 4:1, which is the highest among the caneberry group tested (Table 6) (Yu et al. 2005).

#### Blueberry seed oil (*Vaccinium corymbosum*)

Blueberries contain high concentrations of n-3 fatty acids. ALA is the sole source of the n-3 PUFA and comprises 25.1% of the total fatty acids; which can decrease the ratio of n-6 to n-3 fatty acids (1.7:1). LA is the predominant fatty acid in the blueberry seed oil followed by ALA, oleic, palmitic, and stearic acids (Table 6) (Yu et al. 2005).

#### Cranberry (*Vaccinum macrocarpon*) seed oil

The amount of ALA is found to be 22.3–35% in cranberry seed oil. The ratio of n-6 to n-3 fatty acids is low between 1.2:1 and 2:1 (Table 6) (Parry and Yu 2004; Yu et al. 2005).

#### Sea Buckthorn (*Hippophae rhamnoides* L.) seed oil

The Sea Buckthorn is rich in nutrients. *Hippophae rhamnoides*, *L. sinensis*, and *H. rhamnoides* are subspecies of *H. rhamnoides* L. They contain relatively high percentages of ALA, GLA, and oleic acid. Seed oil samples also have an n-6 to n-3 fatty acid ratio of 2:1 (Table 6). Other fatty acid constituents include palmitic, stearic, and vaccenic (18:1n-7) acids (Yu et al. 2005).

#### Basil (*Ocimum* sp.) seed oil

With regard to ALA, palmitic, and stearic acids, basil species have fatty acid profile similar to that of flax seed oil. Flax and basil seed oils have 52% and 57.4–62.5% ALA, respectively. The n-6 to n-3 fatty acid ratio of the flax seed oil is 1:3.2, while the ratio is 1:1.6–1:3.6 for basil seed oils (Angers et al. 1996). The fatty acid profile of basil is shown in Table 6.

#### Hemp (*Cannabis sativa*) seed oil

ALA, GLA (18:3n-6), and LA constitute 15.1–19.4%, up to 3.6% and 53.4–60.0% of total fatty acids of hemp seed oil (Table 6). Linoleic acid is the most predominant fatty acid followed by ALA, oleic, palmitic, GLA, and stearic acids. Other fatty acids including eicosadienoic, arachidic (20:0), and behenic (22:0) acids are also detected in small quantities (Parry and Yu 2004; Yu et al. 2005).

#### Perilla (*Perilla frutescens*, L. Britton) seed oil

Perilla (*Perilla frutescens*, L. Britton) is a member of the mint family (Lamiaceae or Labiatae). Perilla seed oil contains 51–35% oil, which is similar to flax seed oil fatty acid profile and the amount of PUFA is more than 70% of the total fatty acids. Perilla contains more than 60% ALA (Gunstone et al. 1994), followed by LA and oleic acid (Table 6). The LC-PUFA of perilla is more than that of cotton (Siriamornpun et al. 2006).

#### Chia (*Salvia hispanica* L.) seed oil

*Salvia hispanica* L. (Chia) is an annual herbaceous of the mint family (Labiatae). Chia seeds contain 40–25% oil. The amount of PUFA, especially ALA, is high in chia oil, which is higher than flax oil. Abundance of LA in chia oil is in the second place (26–17%) and PUFA levels in chia oil reach 83%, which is the highest value among edible oils (Table 6). In addition, chia oil has the lowest amount of SFA (Coates and Ayerza 1998; Yu et al. 2005).

#### Camelina (*Camelina sativa*, L. Crantz) seed oil

*Camelina sativa*, L. Crantz belongs to Brassicaceae family. Camelina is a cruciferous plant, which is a member of the mustard family. It has often been labeled as artificial cotton, false flax, desert cotton, and golden delicious (Gold-of-Pleasure). It has fatty acid composition similar to that of flax seed (Table 6). The oil content of camelina is 29–45%. The amount of camelina unsaturated oil is less than that of flax seed, but its oil level is high compared to sunflower and canola oils (Budin et al. 1995; Yu et al. 2005).

#### Lupin (*Lupinus albus* L.) seed oil

Lupins (*Lupinus* spp.) belong to the Genisteae family, Fabaceae, or Leguminosae. The four common lupins are *L. albus* L., *L. angustifolius* L., *L. luteus* L., and *L. mutabilis* L. (Uzun et al. 2007). The oil content in the lupin seed varies from 7.2 to 8.2% (w/w) (Bhardwaj et al. 2004). According to Bhardwaj et al. (2004), white lupin seed oil contains FA in the order of 18:1 > 18:2 > 18:3 > 16:0 > 20:1 > 22:1 > 22:0 > 18:0 > 24:0 > 20:0.

#### Walnut (*Juglans regia*) seed oil

Walnut is a member of the nut family. The fatty acid composition of walnut is 59.7% LA, 13.1% ALA, 15.9% oleic acid, 2.8% stearic, and 8.1% palmitic acid (Venkatachalam and Sathe 2006).

#### Echium oil

Echium seed oil has a unique ratio of *ω*3 to *ω*6 fatty acids among plants (Berti et al. 2007). Echium oil is mainly composed of ALA (30–33%), LA (14–18%), *γ*-linolenic (10–13%), stearidonic (13–15%), oleic (14–17%), and palmitic (6–7%) acids (Table 6). It has been found that *Echium plantagineum* L. contains significant amounts of GLA, ALA, and SDA in seed lipids (Yu et al. 2005).

#### Flax (*Linum usitatissimum* L.) seed oil

Linseed/flax seed is a member of the Linaceae family. ALA includes a high proportion of total PUFAs in flax seed oil. Due to the high content of this unique fatty acid, flax seed oil is used as a nutritional supplement. Soybean and sunflower oils contain lower amounts of SFA compared to flax seed oil. This is while the value is greater than that reported for canola. Fatty acid composition of flax oil is summarized in Table 6 (Gunstone et al. 1994; Hassan-Zadeh et al. 2008; Namazi et al. 2011).

#### Canola (*Brassica napus* L.) seed oil

Rapeseed is a member of the Cruciferae family. Canola oil was for the first time produced in Canada from the seeds of *Brassica napus* and *Brassica rapa*. The oil has low amounts of erucic acid (<2%) and glucosinolates (<30 *μ*mol) and high amount of oleic acid. Canola is another source of ALA. Among all the oils, canola oil contains the lowest amount of SFA. The fatty acid composition of canola is summarized in Table 6 (Przybylski et al. 2005).

#### Soybean (*Glycine max*) seed oil

Soybean is the most important oilseed produced in the world. The presence of relatively high amounts of ALA in soybean oil makes it sensitive to oxidation. The fatty acid composition of soybean is shown in Table 6 (Yu et al. 2005).

#### Malaysian rubber (*Hevea brasiliensis* (Kunth. Muell.)) seed oil (RSO)

The fatty acid composition of rubber seed oil (RSO) comprises of saturated FA (19.12 ± 0.28%) such as palmitic (8.56 ± 0.07%) and stearic (10.56 ± 0.02%) acids and unsaturated FA (79.45 ± 0.31%) such as oleic (22.95 ± 0.15%), linoleic (37.28 ± 0.10%), and linolenic (19.22 ± 0.21%) acids (Table 6) (Salimon and Abdullah 2009). ALA amount in this plant is higher than in canola, rose fruit, soybean, and walnut.

#### Rose fruit seed oil

Major fatty acids present in the rose species seed oil have been characterized as LA (45.38–54.58%), ALA (13.67–24.75%), oleic acid (11.97–21.08%), and palmitic acid (6.54–12.97%) (Table 6). The essential ratio (*ω*6:*ω*3) varies from 1.8:1 to 3.4:1 and oil content in seeds range from 1.3 to 9.0% in different sections (Sharma et al. 2012).

### *γ*-Linolenic acid

GLA is an important unsaturated fatty acid. It is the precursor for AA biosynthesis which is a precursor for prostaglandin formation. Recently, GLA has been recognized for its potential health benefits in the prevention and treatment of cardiovascular disorders (Ratnayake et al. 1989; Yaniv et al. 1989). The presence of significant levels of essential GLA in plants is rare and the most commercially important sources of this fatty acid are seeds of evening primrose (*Oenothera biennis*) with 8–10% (w/w) GLA, borage seeds (*Borago officinalis*) containing 24–25% (w/w) GLA, echium oil with 11.9% GLA at maximum (Berti et al. 2007), and blackcurrant seeds (*Ribes nigrum*) and other *Ribes* species, which contain 16–17% (w/w) GLA (Lawson and Hughes 1988). Although a number of filamentous fungi of the class *Zygomycetes* accumulate large amounts of oil, they tend to have a low GLA content and, conversely, those with high GLA content have only low oil levels. Some of them give a satisfactory overall GLA content in biomass (about 4%) that ensures a relatively high oil yield (more than 20% in biomass) with appropriate concentration of GLA (20–25%) (Certik and Shimizu 1999).

Another practical source of GLA is the microalgae *Spirulina platensis*, which is sold as a nutritional food in several countries, but its effectiveness in producing GLA is not as high as Zygomycetes (Cohen and Heimer 1992).

#### Black currant and other ribe seed oils

Blackcurrant (*Ribes nigrum*) is cultivated for its berries. Blackcurrant seed oil is an excellent dietary source of both GLA and ALA. GLA comprises 12–25% of the total fatty acids, whereas ALA constitutes 10–13% of total fatty acids (Table 7). The GLA concentration is found in other *Ribes* species, including *R. grossularia* (red-black gooseberries), *R. grossularia* (yellow gooseberries), *R. nigrum* (blackcurrants), *R. rubrum* (redcurrants), *R. nigrum*, and *R. hirtellum* (jostaberries). In a study, it was found that among the samples tested, blackcurrant seed oil had the highest level of GLA (Lister et al. 2002; Ruiz Del Castillo et al. 2002, 2004).

**Table 7:** Fatty acid compositions (g fatty acid/100-g oil) of oils with relatively high concentrations of *γ*-linolenic acid (18:3 n−6).

Fatty acid	Blackcurrant	Evining primrose (*Oenothera* spp.)	Evining primrose (*Oenothera biennis*)	Evining primrose (*Oenothera lamarck iana*)	Echium oil	Hemp seed oil	Borage seed oil	Fungi (Mucor)
16:0	6.0–6.3	7–10	9.1	5.8–7.2	6.2	6–9	10–11	9–12
18:0	1.3–1.6	1.5–3.5	3.1	1.5–3.1	3.8	2–3	3.5–4.54	1–2
18:1	8.9–9.6	6–11	17.7	9.2–20.1	16.9	10–16	16–20	20–40
18:2n-6	42.7–43.5	65–80	64.3	62.0–74.6	19.1	50–70	35–38	1–20
18:3n-6	22.0–24.6	8–14	4.9	5.5–9.6	10.5	1–6	17–28	20–40
18:3n-3	10.0–11.5	ND	Trace	ND	29.4	15–25	1	0
	Yu et al. (2005)	Yu et al. (2005)	Yu et al. (2005)	Yu et al. (2005)	Yu et al. (2005)	Angers et al. (1996), Yu et al. (2005)	Sprecher et al. (1999), Yu et al. (2005)	Certik and Shimizu (1999)

#### Evening primrose seed oil

Evening primrose (*Oenothera* spp.) belongs to the Onagraceae family. Evening primrose seed oil is a natural source of GLA (Hudson 1984). Among 192 evening primrose (*Oenothera* spp.) seed oils, the normal range of GLA concentration is 8–14% and the total fatty acids has a broad range of 2–20% with a median value of 10.4% (Hudson 1984). Linoleic acid normally accounts for 65–80% of the total fatty acids with the median value of 73%, which is as high as that of any known vegetable oil. Another study showed that common evening primrose (*O. biennis*) seed oil from Canada contains 4.9% GLA, along with 64% linoleic acid (Ratnayake et al. 1989; Yaniv et al. 1989). In addition, the growing conditions were found to alter the GLA content in the seed oil. The concentration of GLA is in the range of 5.5–9.6% of the total fatty acid content (Table 7) (Simpson 1993a,b).

#### Borage seed oil

Borage is the member of the Boraginaceae family. Traditionally, borage was cultivated for culinary and medicinal uses, although today its commercial cultivation is mainly for using it as an oilseed. The seed oil content is between 26% and 38%. The seed oil is a desirable source of GLA. Borage is the highest known plant-based source of the fatty acid (17–28%) (Eskin 2008). Fatty acid composition of the seed oil is palmitic acid (10–11%), stearic acid (3.5–4.5%), oleic acid (16–20%), GLA (17–28%), linoleic acid (35–38%), eicosenoic acid (3.5–5.5%), erucic acid (1.5–3.5%), and nervonic acid (1.5%) (Table 7). The oil is often sold as “starflower oil” or “borage oil” for use as a GLA supplement, although healthy adults will typically produce enough GLA through dietary linoleic acid (Yu et al. 2005).

### Stearidonic acid

Stearidonic acid is an uncommon fatty acid in higher plants, but very important in human nutrition as SDA is an intermediate in the conversion biosynthesis of ALA to EPA and DHA. Stearidonic acid is found in the oil obtained from genus Echium (Boraginaceae). *Echium plantagineum* L., *Echium plantagineum*, and *Echium vulgare* L. contain 9–16% SDA, while hemp seed (*Cannabis sativa* L., Cannabaceae) contains 2–3% (Callaway et al. 1996), and blackcurrant seed (*Ribes nigrum* L., Grossulariaceae) has about 2% of the fatty acid (Clough 1993; Berti et al. 2007).

## Conclusion

In this review, we investigated the nutritional sources of LC-PUFA with an emphasis on plant sources. Main food sources of *ω*3 LC-PUFA are aquatic species including fishes, shrimps, prawns, crabs, shellfishes, and algae but some problems limit their consumption which include (1) teratogen, carcinogen, and mutagen contaminants including DDT and dioxin-like polychlorinated biphenyls; (2) noncarcinogen contaminants such as methyl mercury, heavy metals (Pb, Cr, Hg, Cd, and As), and antibiotics. Hence there is the need to investigate other sources of LC-PUFA. Herbs, spices, and fruit seeds are good sources of LC-PUFA.
